# Acute Paralytic Disease among Domestic Animals Coincident with Epidemic Poliomyelitis

**Published:** 1917-09

**Authors:** 


					﻿.	q_i	q-i
•	1	O	O	-!
W	•
gw^ •	o	<
.2 s -	•	© £XH, 3	2	u
^3*	■ >	o a £ j'gS *g _• £	3
£?’©	"	S	• PC 3 . s	g p	5 S	< -t-'
O -©	|-jj ^ *.	""	-	. o P9 •	O ® I	U oi
i	il>ig| oll^i so-.g ?Jk
I 5S^£§ c^^pcc
pH	'	X	~	p rP	©	<5	©	£3	2
W P? 2	P " '2	-	42	©	' p
PS	C^=	«	P	2 «i	h	"	3
■p	re re	P	©	3	® £	J®	g	Ji	. P
LP	Ph P	.p	7 Ji O	O -p	Ph
£	<	i p §	.2 -	3	>	°
5	* .S’	=	*	» «•*“§ £ § ■? S
T5	__,	® ©	73 O	42P-V©©^P^-'	Xr
.P	- S	P -	p©©pP©©re	«£
©	3	®	” •	©	-	g 'a re re pi re •©	_»2
P	o S< x pd ct	„	© -Q	“	o	re
•<-<	S?	O 2 P ®	C 5 X 4-	■•" c *	< ?
O -	©	r,- -g g	.P	-©	b£	—	O	6 P	©	P	X	.	>. bt
O ~	ip	X rrt M M	-P	P	O	O	$? ®	72	4>	g	X	-O O
• -2	re	p p ~ ®	©	©	2	74	- p:	-p	*p	K	p	ts
'P	3 x’ —	S	r/T S"~~2	45	2 X _P O ©
gp	g	o§>3|^	|	2	|s	So-g©	8	©	|x”J
•g p	3	-p ’© .go	g	Ph	p	g	re	*>	O ©	O	©	pp	o	® an
2 bo be 5f re	be	%	o	be %	n5	x	re	& o
,.	p	P p o . re	r?	re	.£ s	re ’22 p re	p 2 re	>> p a
i	*	aiil.s	?	J	Sg	.s^sJ	*»$
§	g>2.re re * -g	-g	75	re	re	to	„	re 75	n	re * ©
»	t5 o x x x .	o	©	o	re	2	g	22 © -	©	re	© ,	75	a..22 to ©-
2. - S ‘S ’S ’x 2?’42	p ’42 Ph'x '42 ’x ’42 • o © ’-<- >- I. x g g
O	>.	i***.	r- <*• rz r-H	cZ "T- **• t-
ty	2 =	g £ S	5	£	?	£	=£:£j££5S°21“»=£^-2
£	o	re re re_.re	©	re	ore .reorerePopre~oo©«®3>pr©
j-	M -	x _i	-	_	r—1	're 'pSo a re ^p^
A	~ *42	~ 4 4	re	4	4	2	44 o 4 o 4 4 re - 4 re 4 o 4 4 o
X-.
®	t'-
W -,	O	Z—x
53 .©	-	c?	1-1
O <-.	T—	O	T-l
X P	x_z	^H	,
zP ®	Ci	O
O _-p	p”	Gt	ci	z-s
o a	re _h C3	z—x	t—1
*42 W	z-~x	be .x o	_ •	t—1 —' i—i
£	L-	-p -P Cl	_ O	rH _ Cl
s 22	2P	©	J—Ct	•	O	’n	Ct	O
2|	1	gp§	s	j§3	33	®	_S	I	6	P	SS
<» p	ct p	2	-	re re	3	S re	©	*42	'—'	• '-*•
■g.2	-g	„ -s	~	=	S*x	o =	“5a	=	S	s	£?“
So	g	g.S§	«	.2	| §	8g	|	“fe	£	J	§	, _J
P-i	g	--'go	c;	pgx	•“ *4	x	c®.et4	o	■’-'	ox
~	9^.22	©	©©22	re	—	o	re	75	’pre
©	©	— © iP 42 re 5-	a 73	re	•-	p	P4 72
i>	O	>	°O	05	O	h4	O<1
Conclusions.
Not to make useless repetition of what has been gone over
in the foregoing, a rather dogmatic statement will be made in
summation of the facts elucidated:
It has been demonstrated that the organism isolated from
the nerve centers of cases of poliomyelitis (including the
“streptococcus” described by Rosenow and others) is a
pleomorphic bacillus of the distemper group, which varies in
characteristics much as the various, supposedly different,
members of the group do from one another. That this polio-
myelitis bacillus could cause paralysis in cats, rabbits and
guinea pigs, and that an accidental passage of a culture
through man gave rise to abortive symptoms of the malady.
That after this last named passage it could produce paralysis
in a rabbit, and a contagious infection of guinea pigs with
nerve center lesions; and, finally, that from the guinea pigs
it (the same bacillus) could produce distemper in cats.
Further it is shown that the organism is saprophytic and
grows well in milk at “summer heat;” and that it resists the
pasteurization process, while contained therein. Also, that it
forms spores, and is a “filter passer.”
Finally, that it seems certain that, while contract cases of
. poliomyelitis may occur, either by direct transmission of the
germ from animal to man, or from man to man, the great
mass of cases which comprise epidemics are most probably
caused by milk borne contagion, and that it seems probable
that we shall have to question our cows as possible “carriers”
of the infection, although, it is, of course, evident that in-
fection could get into milk from other animals, as the cat and
dog, or even man.
’Quoted by Frauenthal and Manning in their book on poliomyelitis.
				

